# An Unusual Skull With Bilateral Multiple Bony Lesions on the Parietal and Frontal Bones of the Superior Surface of the Calvaria: A Case Report

**DOI:** 10.7759/cureus.92862

**Published:** 2025-09-21

**Authors:** Anand Verma, Sayan Biswas, Dibakar Borthakur, Hitesh Gurjar, Seema Singh

**Affiliations:** 1 Anatomy, All India Institute of Medical Sciences, Bhubaneswar, IND; 2 Pediatrics, Jorhat Medical College and Hospital, Jorhat, IND; 3 Anatomy, All India Institute of Medical Sciences, New Delhi, IND; 4 Neurosurgery, All India Institute of Medical Sciences, New Delhi, IND

**Keywords:** calvaria, osteoma, osteosclerosis, polyostotic lesions, skull lesion

## Abstract

Calvarial lesions are often asymptomatic and may be discovered incidentally, such as during osteology class. This report describes a unique case of multiple calvarial lesions in an adult skull, emphasizing diagnostic considerations. Small bony lesions were noted on the superior and lateral calvarial surfaces bilaterally in a male skull. CT imaging revealed multiple flat osteosclerotic lesions predominantly on the outer table of the skull, with no involvement of the inner table or diploic space. These smooth lesions were clustered on the parietal eminences and included discrete lesions on the frontal bone, varying in shape from circular to ovoid with irregular margins. The largest lesion measured 0.98x10.32 mm. While benign conditions like osteomas or exostoses are common, differential diagnoses such as fibrous dysplasia and metastatic lesions should also be considered. This case underscores the importance of thorough imaging and clinical evaluation for accurate diagnosis and management of calvarial lesions.

## Introduction

Calvarial lesions are often asymptomatic and discovered incidentally on MRI or CT or as part of diagnosing other diseases or working up local clinical complaints. Rarely, they present as palpable or symptomatic lumps. Diagnosis relies on age, history, and imaging, with benign lesions being more common than malignant ones [1].

The cranial vault is composed of the frontal, parietal, temporal, and occipital bones, as well as portions of the ethmoid and sphenoid bones. There are two tables, the inner and outer tables, with a diploic space between them. The calvaria can be affected by tissue structures or by the intrusion of head or brain lesions into the cranial cavity. Similar lesions can arise in this area since the base of the skull forms the floor of the cranial cavity; however, certain lesions, such as chordoma and chondrosarcoma, are also distinct to this site [2,3].

Radiological diagnosis differentiates benign tumors with well-defined borders and sclerotic edges from malignant ones with ill-defined borders, wide transition zones, periosteal reactions, and soft tissue components. Malignant lesions, often osteolytic and radiolucent, cause significant bone destruction. Skull lesions may be lytic or sclerotic, single or multiple, and arise from various bone constituents. In this case, multiple skull lesions were observed [1-3].

## Case presentation

**Figure 1:**
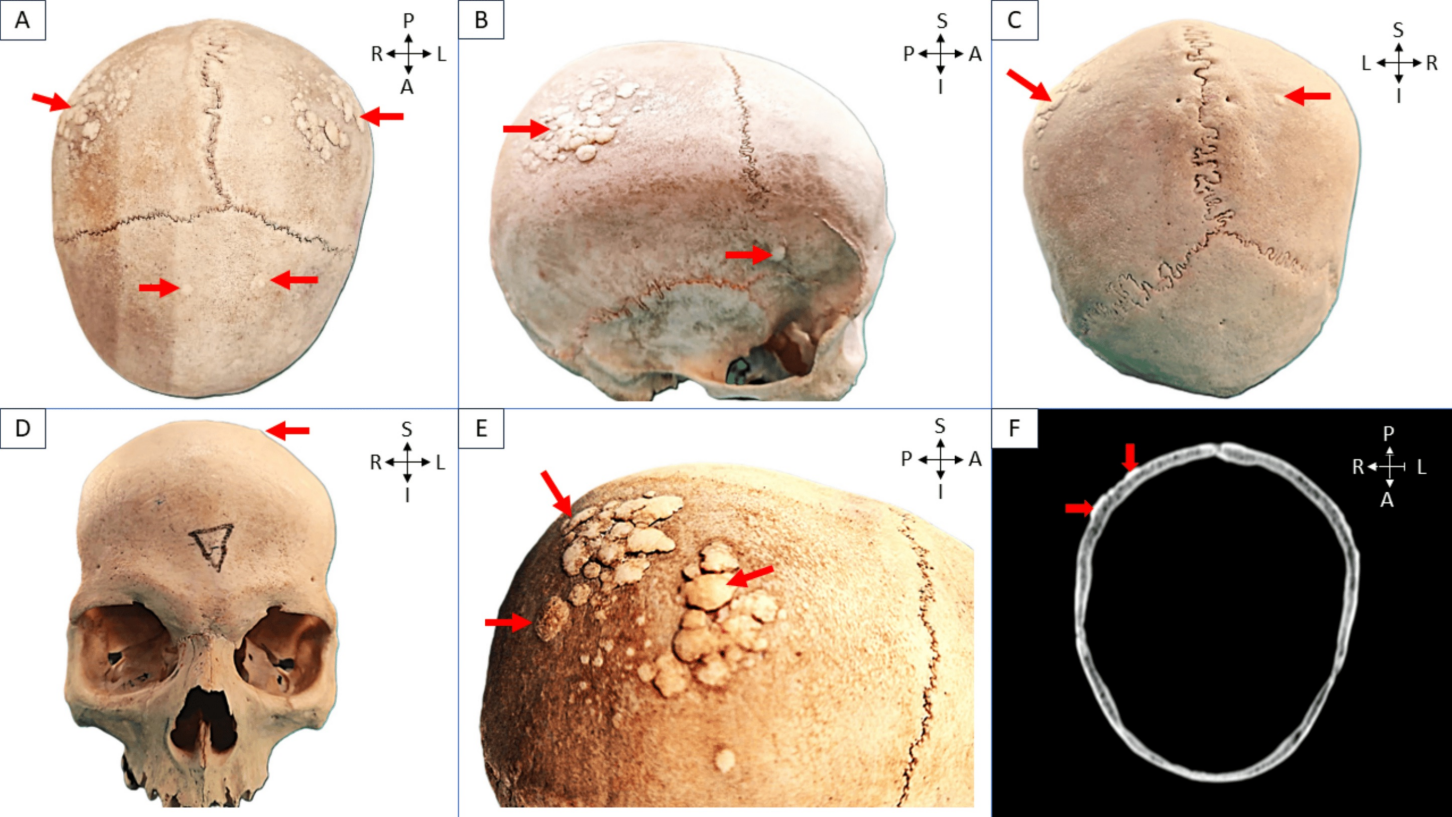
Different views of the skull: (a) superior, (b) lateral, (c) posterior, and (d) anterior, each marked with red arrows indicating the location of the lesions; (e) overlapping lesions on the parietal eminences; (f) CT scan of the skull showing a lesion arising from the outer table, marked with red arrows. Red arrows show the lesions. R, right side; L, left side; A, anterior aspect; P, posterior aspect; S, superior; I, inferior

During a routine osteology class for first-year MBBS students, an intriguing observation was made regarding a skull. It was a 23-year-old male skull in which multiple small bone lesions were observed on the superior and lateral surfaces of the calvaria. These notably flat lesions exhibited a range of shapes from oval to irregular, with diameters varying significantly from 0.98 mm to 10.32 mm. On palpation, the surfaces of the lesions were found to be smooth with irregular margins that remained sharply elevated from the surface. However, the margins of many lesions smaller than 3 mm were observed to be smoothly continuous with the surface. The distribution of these lesions was primarily concentrated on the bilateral parietal eminences, where they appeared as clusters of overlapping lesions. In Figure 1, different views of the skull are presented: (a) superior, (b) lateral, (c) posterior, and (d) anterior, each marked with red arrows to indicate the locations of the lesions, and (e) provides a closer look at the overlapping lesions on the parietal eminences, also highlighted by red arrows. A few isolated lesions were also noted on the frontal bone, though they were less prominent in this region. Interestingly, no such lesions were found on the temporal, occipital, sphenoid, or facial bones, suggesting a specific pattern of occurrence. No lesions were observed in the basal part of the skull.

Closer examination revealed that all the lesions seemed to arise from the outer table of the skull, with no involvement of the inner table or diploic space, as confirmed by CT scan and shown in Figure 1f. Despite these abnormalities, the rest of the skull's anatomical features were unremarkable, showing no signs of deformity or other pathological conditions.

## Discussion

The differential diagnoses considered in this case included osteoma, exostoses, osteoid osteoma, hyperparathyroidism, fibrous dysplasia, metastasis, Langerhans cell histiocytosis (LCH), osseous venous malformation, and meningioma (Table 1). To differentiate bone neoplasms from other disease entities and accurately diagnose them, a comprehensive analysis is essential. This should include a detailed description of the lesions, focusing on their location, extent, number (whether single or multiple), as well as their bilateral or symmetric presentation and morphological characteristics. In certain cases, considering the patient's age can also provide valuable insights, aiding in the differential diagnosis [4].

**Table 1:** Summary of the features of neoplastic and tumor-like lesions of bone.

S. No.	Type	Common Location - Skeleton	Main Radiographic Features
1	Osteoma	Craniofacial bones	Radiodense/sclerotic, outer table
2	Exostoses	Long bones, tubular bones of the hands and feet, pelvic bones and cranial base, facial bones are rarely involved	Sessile or pedunculated bony outgrowths. Continuous with the host bone
3	Osteoid osteoma	Long bones (femur, tibia), hands, feet, vertebrae	Round or oval radiolucent nidus with surrounding sclerosis
4	Hyperparathyroidism	Long bones, pelvis, spine, skull	Granular decalcification (salt-and-pepper appearance), osteosclerosis
5	Fibrous dysplasia	Femur, tibia, craniofacial bones, ribs, pelvic bones	Radiolucent with sclerotic margins to radiopaque. Ground-glass appearance is common.
6	Bone metastasis	Pelvic and thoracic bones, craniofacial and long bones	Osteoblastic, osteolytic, mixed lesions. Variable type of margins
7	Langerhans cell histiocytosis	Skull	Initial lesions appear as osteolytic areas with beveled edges due to uneven inner and outer table involvement
8	Osseous venous	Calvarial lesions	Well-circumscribed intradiploic osteolytic lesion with mild expansion of the outer table and relative sparing of the inner table
9	Meningiomas	Frontoparietal and orbital regions	Sclerotic lesion with associated hyperostosis of the bone, and irregular and spiculated borders
10	Present case	Skull (parietal and frontal bone)	Sessile and multiple flat lesions on the outer table of the skull

An osteoma is a well-organized osteogenic tumor developed from adult bone. This benign bone-forming disorder has no known etiology, and it is still unclear if it has dysplastic or neoplastic roots [5]. On the basis of histologic patterns, osteomas are divided into three types: first, the ivory or compact osteoma, made of mature, dense lamellar bone lacking Haversian canals; second, the developed or cancellous osteoma, which contains trabecular bone and resembles normal bone; and third, the mixed type, which combines features of both. Osteomas are typically an accidental discovery in clinical settings, making it challenging to determine their occurrence. Osteomas are seldom found in the long bones and nearly always arise in the craniofacial skeleton [5,6].

The cranial vault and mandible can develop osteomas, also known as "button osteomas" or "ivory exostoses." These lesions, composed of dense, mature lamellar bone, are relatively rare and typically measure 2-4 cm in diameter. Usually found on the bone's outer table, they have a compact, smooth structure and may exhibit slight undercutting where they join the outer table. While routinely a solitary lesion, multiple osteomas can occur, particularly in cases of Gardner syndrome. The parietal and frontal bones are the most common sites for these lesions. In the present case, multiple smaller lesions were observed, with some overlapping, which contrasts with the well-demarcated lesions seen in button osteoma [5,6].

An osteoid osteoma is a benign bone tumor, accounting for 10-12% of all benign bone tumors, and is often linked to chromosomal abnormalities, particularly on 22q and 17q. It consists of highly vascularized fibrous tissue with osteoblasts forming osteoid or immature bone [6,7]. On radiography, it shows up as a tiny, well-defined, radiolucent nidus that is 1.5-2 cm in size and may include core calcifications. It is encircled by a broad area of bone sclerosis, which can result in cortical thickening and changes in the shape of the bone. It is more commonly observed in males, similar to the current study. However, the lesions were only seen on a detached skull in this instance, despite the fact that they are frequently detected in long bones, hands, feet, and vertebrae [5,6]. Moreover, a CT scan of osteoid osteoma typically shows characteristic vascular grooves radiating from the nidus, which is absent in the present case.

Hyperparathyroidism is characterized by brown tumors, subperiosteal bone resorption, pathological fractures, osteopenia, terminal acrosteolysis, and a "pepper-and-salt" appearance of the skull. While the link between osteosclerotic changes and secondary hyperparathyroidism remains unrecorded, particularly in renal osteodystrophy, in this case, multiple, dense, overlapping bony lesions were observed; however, a CT scan performed to assess for the "salt-and-pepper" skull appearance showed the absence of this feature [8].

Fibrous dysplasia is a benign bone lesion caused by abnormal osteogenesis, leading to intramedullary fibroosseous growth. It can present as monostotic (one bone) or polyostotic (multiple bones) forms. Complications may arise from the replacement of normal bone with fibro-osseous tissue, including compression or fractures of nearby soft tissues, especially neurovascular structures. Monostotic fibrous dysplasia often remains asymptomatic, while the polyostotic form typically manifests in childhood. Adult cases are often discovered incidentally during imaging for unrelated reasons. In this case, a CT scan revealed the absence of intramedullary growth [1,2].

Multiple metastatic skull lesions are frequently associated with adult breast, lung, prostate, kidney, or thyroid malignancies, and with neuroblastoma or sarcomas in children. Such lesions may be solitary or multiple and are commonly osteolytic, whereas prostate cancer may produce osteosclerotic lesions [1]. The lesions extend into the surrounding soft tissues, which contrasts with the present study, where the lesions are limited to the outer table of the cranial vault.

LCH, formerly histiocytosis X, involves abnormal Langerhans cell proliferation in various tissues, with eosinophilic granuloma as the most common form, often affecting the calvarium. On CT, initial lesions appear as osteolytic areas with beveled edges due to uneven inner and outer table involvement, often featuring a central "button sequestrum" of intact bone. These lesions usually lack a sclerotic rim or periosteal reaction, and multiple lesions may coalesce, creating a geographic pattern. They can also extend into soft tissue and beyond the skull, which may later sclerose during healing [1,2].

Osseous venous malformations are benign, slowly developing vascular bone tumors, comprising 2-10% of benign calvarial lesions and 0.2% of all bone tumors. Common in the frontal and parietal bones of individuals in their 40s and 50s, they typically present as small, asymptomatic lesions but can cause discomfort, deformity, or functional issues if near critical structures. A well-defined intradiploic osteolytic lesion is seen on CT, with the inner table mostly spared and the outer table mildly expanded. The typical discovery is a sunburst pattern of trabecular thickening emanating from a common center that is absent in the current case [1,2].

Meningiomas are the most common extra-axial dural tumors in middle-aged and older adults, often causing reactive hyperostosis in adjacent bone, leading to significant skull thickening of the outer table and outward expansion. Rare intraosseous meningiomas, accounting for less than 2% of all meningiomas, typically occur in the frontoparietal and orbital regions. These ectopic tumors may arise from arachnoid cap cell entrapment during skull development or post-trauma. Intraosseous meningiomas have an 11% risk of malignant transformation, higher than the 2% seen in primary intradural meningiomas. CT imaging mostly reveals sclerotic lesions with hyperostosis and irregular, spiculated margins; however, in the case of metastatic meningiomas, osteolytic lesions are observed. Sclerotic intraosseous meningiomas involve the inner table and can project outward from the external surface by destroying the outer table, which, however, was intact in the current study [1,5,9].

## Conclusions

In our case, the presenting features are more in favor of osteoma-related clinical findings, while various differential diagnoses were considered, including osteoid osteoma, hyperparathyroidism, fibrous dysplasia, metastatic disease, Langerhans cell histiocytosis, and intraosseous meningioma. Continued investigation will not only aid in understanding this particular case but also enhance the diagnostic framework for similar presentations in clinical practice, underscoring the importance of meticulous analysis in osteological studies. Further diagnostic work-up, including histological examination, is essential for accurate diagnosis and appropriate management.
